# Visit Characteristics Associated With Pediatric Dental Appointment No-Shows in an Academic Dental Setting

**DOI:** 10.1155/ijod/2114933

**Published:** 2025-07-11

**Authors:** Rubelisa C. G. de Oliveira, Hassan Khalid, Zhaoqi Zhang, Saptarshi Chakraborty, Daniela Benzano, Jessica S. Kruger, Susan C. McKernan

**Affiliations:** ^1^Department of Periodontics and Endodontics, University at Buffalo, Buffalo, New York, USA; ^2^Department of Epidemiology and Environmental Health, University at Buffalo, Buffalo, New York, USA; ^3^Department of Biostatistics, School of Public Health and Health Professions, University at Buffalo, Buffalo, New York, USA; ^4^Department of Epidemiology, School of Medicine, Federal University of Rio Grande do Sul, Porto Alegre, Rio Grande do Sul, Brazil; ^5^Department of Community Health and Health Behavior, School of Public Health and Health Professions, University at Buffalo, Buffalo, New York, USA; ^6^Department of Primary Dental Care, Division of Dental Public Health, School of Dentistry, University of Minnesota, Minneapolis, Minnesota, USA

**Keywords:** appointments, dental care for children, health disparity, no-show, pediatric dentistry, social deprivation

## Abstract

**Objectives:** Dental no-show rates negatively impact oral health outcomes, especially among adolescents. While many factors can be associated with no-show in dental appointments, adolescents' no-show rates are influenced by parents' or caregivers' experiences and decisions. This study aims to investigate patient visit-related characteristics that are associated with failure to attend scheduled dental appointments in an academic institution located in Buffalo, New York (NY), a region impacted by health disparities.

**Methods:** A secondary analysis was performed with multivariables extracted from the electronic health records of individuals aged 0–19 years from 2018 to 2023. Bivariate and multivariate analyses at the visit level were performed to compare show/no-show groups concerning demographics, payor at the time of dental appointment, distance from home to dental facility, and Social Deprivation Index (SDI) scores related to their ZIP codes.

**Results:** A total of 7379 visits were included in the analysis. 14.3% were no-shows. Adolescents aged 12–17 years accounted for the greatest no-show rate (24%) when compared with younger children. Age had an increased likelihood of no-show (OR = 1.13, CI = 1.11–1.15), and social deprivation had a slight protective effect in no-show at dental appointments in this academic institution (OR = 0.98, CI = 0.98–0.99). The great majority of pediatric patients come from socioeconomically deprived areas and live further away from the school.

**Conclusions:** Adolescents are the group at most risk of no-show. Future studies should explore strategies to better understand the barriers related to this lifespan as well as implement interventions that facilitate scheduling as well as prevent no-shows.

## 1. Introduction

No-shows, or failure to keep scheduled dental appointments, negatively impact oral health outcomes for patients. No-shows also burden the dental care system, leading to a loss of clinical productivity, poor treatment outcomes, and ultimately the loss of revenue [1]. In the academic setting, patient no-shows also adversely impact the learning process for dental students and residents.

There are multiple factors associated with missed dental appointments. Limited research has found that Black/non-Hispanic and Hispanic individuals, compared to their White/non-Hispanic counterparts, had an increased likelihood of no-shows for dental appointments [2]. Travel distance and other transportation-related issues have also been reported as enabling factors in a person's decision to see a dental care provider [3].

For children and adolescents, parental attitudes add additional barriers for keeping scheduled appointments [4–6]. Parental experiences of fear and anxiety related to dental treatment can have a significant impact on how parents approach their child's dental care; they may avoid dental appointments to shield their child from similar distress [6]. Communication barriers, particularly for parents whose primary language is not English, can further exacerbate concerns about dental treatment. Parents with a primary language other than English have shown difficulty in insuring their children because of a lack of knowledge about insurance programs, immigration issues, and difficulties navigating the healthcare system [7]. Additionally, children from low-income households with public insurance were reported to be more likely to no-show than children from high-income household and privately insured [8, 9]. This highlights the role of not only individual factors but also broader familial and geographic-level determinants in shaping dental care behaviors.

To account for these influences, this study uses the Social Deprivation Index (SDI) developed by the Robert Graham Center as a proxy for socioeconomic status (SES) [10]. The SDI is a validated composite measure that reflects multiple dimensions of social disadvantage, including income, education, employment, housing, household characteristics, and access to transportation. Although SDI can be calculated at different geographic levels (such as census tract or county), in this study we computed SDI at the Zip Code Tabulation Area (ZCTA) level, aligning with the availability of patient residential zip code data. By mapping each patient's zip code to the corresponding ZCTA SDI score, we aim to account for the complex interplay of conditions that contribute to disparities in dental attendance patterns, offering a more accurate context for interpreting our findings.

Missing a scheduled dental visit represents an opportunity cost that has negative implications for individual and population health. Dental problems, if left untreated, can escalate into more serious health issues [11, 12]. A missed dental appointment for a child or adolescent means a lost opportunity to establish a dental home. According to Healthy People 2030, regular dental visits during childhood and adolescence are essential for maintaining oral health and preventing dental issues like caries [13]. Consequently, the effort to better understand and reduce no-show rates in dental appointments is not only a matter of improving scheduling efficiency but also a critical strategy in promoting oral health equity and achieving Healthy People 2030 objectives to increase the proportion of low-income youth who have a preventive dental visit.

Few studies have investigated the demographic and visit-related characteristics of young individuals who depend on academic institutions for dental services in the United States [2, 8, 14–17]. A prospective study in Iowa compared the appointment-keeping behavior of children aged 18 and younger across different types of pediatric dental facilities, including a private pediatric dental office, a public health dental clinic, and a university-based clinic [16]. Overall, Medicaid-enrolled children had a significantly greater proportion of failed appointments (defined as failing to cancel or show up for a scheduled appointment) than other children (24% vs. 7%, respectively; *p* < 0.0001) [16]. Interestingly, the dental school clinic had the lowest rate of failed pediatric appointments (7%) compared to the public health clinic (10%) and the private dental office (14%). However, insurance coverage and clinic type were the only covariates that were examined [16]. Two other studies tested the impact of the implementation of text messaging systems based on surveys showing different outcomes with this strategy [14, 17]. Two additional U.S. studies on no-show behavior relied on retrospective data extracted from available management practice software [2, 8]. Both showed that multifactorial issues are associated with no-shows for dental appointments, and access to continuous dental care for children needs to be improved.

Due to this scarcity of research and eminent need for innovative solutions, the purpose of this study is to investigate patient visit-related characteristics that are associated with failure to attend scheduled dental appointments in an academic institution located in Buffalo, New York (NY), a region impacted by health disparities. By the time of this study, it had also been reported that residents of the City of Buffalo had been without fluoridated water since 2015. Although fluoridation officially resumed in September 2024, the long-term effects of this prolonged absence remain unclear, particularly with respect to its impact on the oral health of adolescents, a group especially susceptible to dental caries during developmental years [18].

## 2. Methods

A retrospective exploratory chart review of dental visits of patients aged 0–19 years seeking treatment at the School of Dental Medicine at the University at Buffalo (SDMUB), NY, during 5 years (2018–2023) was performed. A de-identified and coded dataset extracted from AxiUm electronic dental records (EDR) was built, with multiple explanatory variables on individual patients visiting the dental clinics to identify various patient characteristics that have been associated with no-shows in dental appointments. This cross-sectional study was submitted and approved by the Institutional Board of Research Committee, University at Buffalo (#STUDY00007027).

The inclusion criteria were young individuals aged 0–19 years of either sex and any race/ethnicity, including individuals from populations with health disparities as defined using NIH criteria [19] seeking treatment at the dental clinics at SDMUB.

Individuals met inclusion criteria when they had at least one scheduled follow-up appointment subsequent to a routine visit attended within 5 years. Duplicate events, provider-initiated or administrative cancelations, and records with missing address information that could not give access to individual ZIP codes were excluded.

### 2.1. Data Collection, Explanatory Variables, and Outcome Variable Definitions

For this study, a no-show was defined as a previously scheduled dental appointment that the patient did not attend and for which there was no prior notice of cancelation, or the appointment was canceled less than 24 h without being rescheduled. In contrast, appointments that were canceled more than 24 h in advance or that were rescheduled at the time of cancelation were not considered no-shows. This definition was used to distinguish true missed appointments from cancelations that allowed for schedule adjustments.

Visit characteristics were summarized by show versus no-show rates for all variables cataloged in the dataset. Analysis was performed to compare scheduled visits concerning demographic variables, such as age, gender, ethnicity, race, and other explanatory variables, including travel distance. Travel distance was defined here as the distance in miles calculated from their home address to the dental clinic building, and SDI scores [10] related to their ZIP codes, payor at the time of dental appointment, and how the show/no-show rates were impacted during COVID-19 pandemic. Analyses controlled for the effects of pandemic-related dental office closures.

All AxiUm Current Dental Terminology (CDT) codes (diagnostics, preventive, restorative, endodontics, periodontics, removable and fixed prosthodontics, surgery, and orthodontics procedures) were included. To control for the impacts of the COVID-19 pandemic in 2020, we created an indicator variable to identify appointments that were scheduled from 03/16/2020 to 12/31/2020. This encompasses the period when the SDMUB dental clinics were closed due to the 2020 federal public health emergency, or when appointments were limited to emergencies or urgent dental conditions.

Payor status was categorized as public dental benefits or self-pay. Public dental benefits in NY state include Medicaid, CHIP, and the NY essential plan (covering households with income at 138%–200% of the federal poverty level). SMDUB does not accept private insurance.

To have a better sense of the income level or SES of the patient, we used the patient's ZIP code and applied the SDI [10] at the ZCTA. SDI is “a composite measure of area-level deprivation based on the percent living in poverty; percent with less than 12 years of education, percent in single parent households, the percentage living in rented housing units, percent living in overcrowded housing units, percent in households without a car, and percentage nonemployed for the population 16–64 years.” [10] SDI scores range from 1 to 100. Higher SDI scores indicate greater social deprivation and lower income levels and have been strongly and consistently associated with poor health and healthcare outcomes. SDI has been studied more in medicine [20–23]. No studies to date have examined this index in relationship to dental appointment-keeping behaviors.

We assessed patient travel distance based on the Vincenty method in the geosphere R package [24] by calculating the straight-line distance between the geographic centroids of the patient and the SDMUB's ZIP codes, which assumes the earth is elliptical [25].

### 2.2. Statistical Analysis

Continuous variables were summarized using medians and interquartile range (IQR). Categorical variables were summarized as frequencies and percentages. Age, commute miles, and SDI scores were analyzed as continuous and categorical variables. Travel distances were categorized as ≤20 miles, 20–50 miles, and >50 miles. Travel distances under 20 miles are considered a short commute by the US Census Bureau [26]. Travel distances >50 miles are considered long commutes.

To identify the explanatory variables/factors most strongly associated with no-shows in dental visits in this academic institution, we considered two sets of inferential statistical association analyses. In the first, we compared the overall differences in these explanatory variables values for the show and no-show groups using Pearson's Chi-squared and Fisher's exact tests for comparing categorical explanatory variables and the Mann–Whitney test for comparing continuous explanatory variables.

Next, a multivariable analysis was performed using a logistic regression model on show/no-show status with main effect terms for all the explanatory variables, including travel distances, and SDI scores. We also tested for interactions between patient age and gender, travel distances, and SDI scores. Due to the high percentage of missing data on race and ethnicity, as well as individuals with nonreported payor status, these variables were excluded from the multivariable analysis. To ensure stable estimation of the logistic regression model parameters, particularly given the voluminousness of the categorical levels of the explanatory variables and their interaction terms, we used a weakly informative t-prior for the regression coefficients and obtained the consequent maximum a posteriori estimates using an approximate EM algorithm [27]. Statistical inferences were then made using asymptotic approximate normality of the resulting estimates (the total sample size is *n* = 7379) [28]. All computations were done in statistical software R v4.2.0 (R Foundation) and SPSS version 20.0 utilizing diverse packages.

## 3. Results

**Figure 1:**
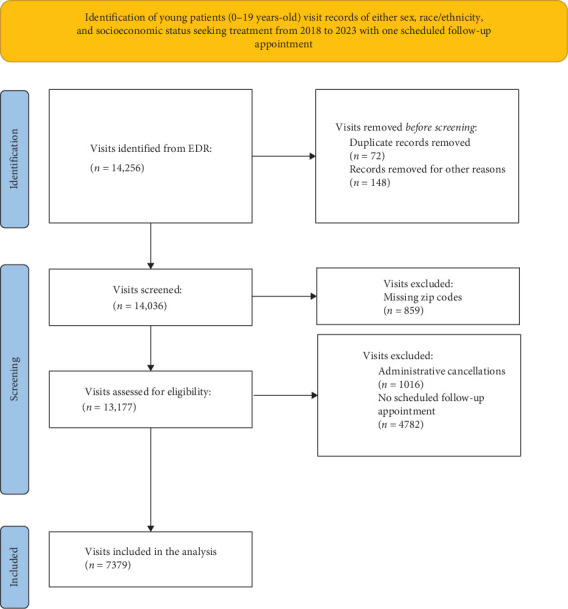
Flowchart of the sample.

Patient and visit characteristics related to show and no-show rates are described in Table 1. A total of 7379 visits (825 individuals) were included (Figure 1). Preventive procedures were the most prevalent among the investigated visits (64.2%), followed by no-shows (14.3%), restorative (12%), orthodontics (4.8%), and advanced procedures (periodontics, endodontics, and surgical procedures) (4.1%). Descriptive analysis and visit characteristics across show and no-show groups are presented in Table 1.

Patients were grouped into five age subgroups. Adolescents aged 12–17 years showed the highest prevalence of no-shows in dental appointments (24%) when compared with other age groups (*p*  < 0.001). There was no statistically significant difference in no-show rates between males and females. A majority of patients did not include information on race (unknown/missing = 61.7%) and/or ethnicity (unknown/missing = 68.1%). Among those who self-reported their race/ethnicity, 22% identified themselves as White, 10.8% as Black or African American, 2.9% as Asian, and 2.5% as other. Self-reported Hispanics or Latinos represented 5.6% of our sample.

Appointments tied to self-pay represented a larger proportion of no-shows when compared to appointments tied to public insurance (16% vs. 11%, respectively; *p*  < 0.001).

In the univariate analysis, there was a statistically significant difference within the travel distance categories when show/no-show groups were compared. Interestingly, this comparison between groups showed that individuals facing a travel distance greater than 50 miles presented the lower no-show rate (6.4%), followed by individuals coming from less than 20 miles (19.0%) and those coming from a moderate distance, 20.1–50 miles, (36.0%).

This difference was more evident when we looked closely at the two largest groups in a number of dental visits: 0–20 miles and ≥50.1 miles. Approximately one in five scheduled visits for patients with travel distances <20 miles resulted in a no-show. Conversely, 6.4% of scheduled visits for patients with travel distances ≥50.1 miles resulted in a no-show. Both groups together represent more than 94% of the dental visits investigated. Appointments for individuals living 20.1–50 miles away demonstrated the highest percentage of no-shows (36.0%).

Overall, more than 57% of visits were from individuals with SDI scores between 78.1 and 100, meaning that most of the individuals come from socioeconomically deprived areas. Individuals living in an area with SDI scores of 1–19 represented 8.5% of our sample and showed the highest no-show rates (37.2%) when compared to individuals with SDI scores >19.1. There was a statistically significant difference within the SDI score categories when show/no-show groups were compared (*p*  < 0.001).

The greatest proportion of visits was self-pay (52.1%), which demonstrated a significantly greater proportion of no-shows compared to visits covered by public insurance benefits (Table 1). The multivariable analyses identified factors that were significantly associated with the likelihood that an individual had a no-show at this academic institution. Adjusted ORs (95% CIs) associated with no-shows in dental appointments in this academic institution are presented in Table 2. The logistic regression showed that age, SDI score, and pandemic were significantly associated with no-show rates when other explanatory variables were controlled. Gender, travel distance, and payor were not significantly associated with no-show rates. Interactions between age, gender, travel distances, and SDI scores did not show any statistically significant association.

As the patient's age increased, the odds of not showing up for a scheduled appointment increased by 13% with each additional year of the patient's age. Controlling for all other factors in the model, SDI score and pandemic were negatively associated with odds of no-shows (OR = 0.98 and 3.15, respectively; *p*  < 0.001). People from areas with higher SDI scores (i.e., residing in more socially deprived areas) had greater odds of attending a scheduled appointment within this academic institution. There were decreased odds of a no-show during the pandemic. Although the overall proportion of appointment no-shows increased during the pandemic when compared with regular times (show = 203 [57%]; no-show = 153 [43%]), the adjusted odds of an individual attending a scheduled appointment during the pandemic were about three times as high compared to an appointment scheduled outside the pandemic.

## 4. Discussion

Our study observed a 14.3% no-show rate at this academic institution located in Western NY, which is within the reported nationwide prevalence of dental appointment no-show range from 5% to 38% [2, 8, 14–17]. Our data showed that this academic institution provides dental care for populations coming from areas of social deprivation, with more than 57% of pediatric patients seen between 2018 and 2023 coming from households in a ZIP code with high social deprivation (i.e., SDI scores 78.1–100). Some variables related to social determinants of health, such as demographics, social deprivation, commute miles, and dental payor, were significantly associated with no-show rates in univariate analysis. However, commute miles and payor did not remain statistically significant when other factors were controlled for in the multivariable analysis. In the adjusted model, age, SDI score, and the pandemic period had an impact on the no-show rates among the young individuals seen at this academic institution.

Age was negatively associated with dental visit no-shows within this academic institution. For each additional year of age, the odds of a failed appointment increased by 13%. Adolescents (i.e., aged 12–19 years old) had a greater prevalence of no-shows than the other age subgroups. There were no statistically significant differences observed between genders. These findings are similar to the majority of studies on factors related to no-shows in overall healthcare appointments. Age generally has an inverse relationship with the probability of missing appointments, with older children more likely to miss their scheduled dental visits [1, 29–31]. While gender does not typically emerge as a statistically significant factor in most studies, reports are suggesting that males might be more prone to no-shows in healthcare appointments compared to females [1, 8, 9, 32–34].

Interestingly, our analysis showed that individuals from areas with the greatest social deprivation (i.e., SDI > 38) were more likely to attend their dental visits than individuals coming from decreased social deprivation, and low SDI scores (<38) (Table 1). This contrasts with the findings of Discepolo et al. [2], who reported that children residing in areas with greater social vulnerability indices (SVIs) were slightly more likely to miss a scheduled appointment than children from lower SVI areas. One potential explanation for our differing result is that children from areas with less social deprivation may have more resources or opportunities to attend their dental appointments than those from low-income households [3].

For this specific Western NY region, few dental offices accept Medicaid insurance [35]. A local report led by the Community Health Foundation of Western and Central NY observed that the majority of local dentists who participated in the study (67%) did not accept public insurance, and only seven (19%) were located in areas of poverty [35]. Thus, parents and caregivers with limited access to dental care may recognize the limited access and emphasize on keeping their children's appointments.

Previous literature has suggested that increased distance traveled is related to lower utilization of dental visits [36]. However, we did not find an association between travel distances and dental no-shows within our dental school when controlling for other variables. For instance, the individuals coming from further away (more than 50 miles) showed decreased prevalence of no-shows when compared with those living nearby (less than 20 miles). McKernan et al. [3] also observed in their adjusted model that travel distance was not significantly associated with the likelihood of dental care utilization; however, other transportation-related factors were more important.

Parents or caregivers willing to drive further distances also may live in geographically remote areas, where a higher percentage of people live in dental provider shortage areas [37–39]. The dental appointment for those families might be more of a priority than for those who can have more options of access to dental care.

Although self-pay visits in this study had a greater prevalence of no-shows compared with visits covered by public insurance, payor status was not statistically significantly associated with the likelihood of no-shows in multivariable analysis. In this population, “self-pay” includes patients with private dental benefits and individuals who lack insurance coverage, and people who have private benefits. Even though there have been improvements in children's utilization of dental care over the past 20 years driven by increased public insurance enrollment, [40–42] this does not necessarily mean that affordability is not an issue anymore [43]. Dental insurance plans typically cost more for U.S. families in general when compared with medical care due to more cost-sharing mechanisms in place in dental insurance, including higher rates of coinsurance and annual maximum benefit limits [43]. Thus, financial issues are still a burden for dental care utilization, but other multiple factors impact a person's decision to see or not see a dental provider, such as the need due to the lack of availability of dental services near the residential address or being diseased or in pain, as described by the Andersen's model in 1968 [44], and still up-to-date.

This cross-sectional study has some limitations. First, the EHR data were collected from a single academic institution, which may limit the generalizability of our findings to other settings, such as private practices or community clinics with different patient populations and operational structures. Second, the study relied on retrospective EHR data, which may introduce bias due to variability in documentation practices across staff and providers. The reliance on preexisting administrative data also limits the ability to draw casual inferences. Third, patient race and ethnicity were not consistently recorded, preventing analysis of the impact of sociodemographic characteristics on no-shows. Additionally, while the SDI is a validated composite measure of social disadvantage, it was applied at the ZCTA level rather than a more granular scale, possibly masking within area variation. Previous research has shown that ZIP code-level aggregation can mask localized patterns of deprivation [2]. Fourth, this study did not include qualitative methods, such as patient interviews or surveys. As a result, it could not capture nuanced individual-level factors, such as fear, competing responsibilities, or cultural beliefs that may contribute to a no-show. Last, we lacked access to detailed information on provider-initiated or administrative cancelations, referral sources, and specific dental needs of the patients included in this dataset, which may have further contextualized patient attendance patterns.

Despite these limitations, the study offers valuable insights into the characteristics of dental appointments within an academic institution, a setting that remains relatively underexplored in dental research. By focusing on this unique environment, the study sheds light on dental care utilization patterns among populations that often rely on these institutions as their primary source of care, especially those experiencing social deprivation. Additionally, this study is original by examining youth dental care utilization, as well as by including the SDI as an area-level social risk factor. This approach not only highlights the specific needs of children and adolescents but also has the potential to inform more effective strategies for improving dental health among socioeconomically disadvantaged populations.

To overcome the limitations of secondary data analysis, future investigations are needed. Incorporating findings from this study, our research group plans to survey parents and caregivers to better understand drivers of dental attendance. Specifically, social determinants of health for dental care access, oral health literacy, anxiety, providers' empathy, and dental care satisfaction will be included in this upcoming research phase.

## 5. Conclusion

This study showed that age and area-level social deprivation were associated with no-shows in dental appointments in an academic institution. Adolescents were more likely to no-show than younger children at SDMUB. In addition, the pediatric patients seen in this dental school who came more from areas of greater social deprivation, and further distances were less likely to no-show for dental appointments. Overall, our findings have the potential to generate strategies to improve patient dental care utilization within this academic institution. SDMUB's IT Department, alongside the reception and wellness center, will develop an algorithm-informed application to add alerts related to those visit characteristics with an increased likelihood of no-show, with the ultimate goal to facilitate scheduling as well as prevent no-shows.

## Figures and Tables

**Table 1:** Patient demographic and visit characteristics by attendance among young individuals scheduled in the academic institution between 2018 and 2023 (overall no-show = 14.3%).

Characteristic	Overall *N* (%)	Show *N* = 6322^a^	No-show *N* = 1057^a^	*p*-Value^b^
Age	—	—	—	<0.001
Median (IQR)	—	9.9 (7.2, 14.5)	14.2 (11.2, 16.2)	—
Age grouped	—	—	—	<0.001
0–7	2252 (30.5%)	2099 (93.2%)	153 (6.8%)	—
8–11	1853 (25.1%)	1698 (92%)	155 (8.4%)	—
12–17	2675 (36.2%)	2042 (76%)	633 (24%)	—
18 and above	599 (8.2%)	483 (81%)	116 (19%)	—
Gender	—	—	—	0.14
Female	4097 (55.5%)	3488 (85%)	609 (15%)	—
Male	3282 (44.5%)	2834 (86%)	448 (14%)	—
Race	—	—	—	<0.001
Missing	4556 (61.7%)	3663 (80%)	893 (20%)	—
White	1624 (22%)	1520 (94%)	104 (6.4%)	—
Black or African American	797 (10.8%)	764 (96%)	33 (4.1%)	—
Asian	217 (2.9%)	201 (93%)	16 (7.4%)	—
Other	185 (2.5%)	174 (94%)	11 (5.9%)	—
Ethnicity	—	—	—	<0.001
Missing	5028 (68.1%)	4092 (81%)	936 (19%)	—
Hispanic or Latino	412 (5.6%)	397 (96%)	15 (3.6%)	—
Not Hispanic or Latino	1939 (26.3%)	1833 (95%)	106 (5.5%)	—
Travel distance (mile)	—	—	—	<0.001
Median (IQR)	19 (5, 72)	33 (5, 80)	10 (4, 25)	—
Travel distance grouped	—	—	—	<0.001
0–20	3741 (50.7%)	3031 (81%)	710 (19%)	—
20.1–50	383 (5.2%)	245 (64%)	138 (36%)	—
≥50.1	3255 (44.1%)	3046 (93.6%)	209 (6.4%)	—
SDI score	—	—	—	<0.001
Median (IQR)	—	82 (48, 91)	51 (28, 89)	—
SDI score grouped	—	—	—	<0.001
1–19	627 (8.5%)	394 (62.8%)	233 (37.2%)	—
19.1–38	979 (13.3%)	764 (78%)	215 (22%)	—
38.1–58	868 (11.7%)	746 (85.9%)	122 (14.1%)	—
58.1–78	704 (9.5%)	642 (91.2%)	62 (8.8%)	—
78.1–100	4201 (57%)	3776 (89.9%)	425 (10.1%)	—
Payor	—	—	—	<0.001
Not reported	65 (0.9%)	0 (0%)	65 (100%)	—
Public	3472 (47.1%)	3082 (89%)	390 (11%)	—
Self-pay	3842 (52.1%)	3240 (84%)	602 (16%)	—

^a^ *N* (%).

^b^Pearson's Chi-squared test; Wilcoxon rank sum test; Fisher's exact test for count data with simulated *p*-value (based on 10,000 replicates).

**Table 2:** Multivariate analysis: adjusted ORs (95% CIs) associated with no-show in dental appointments in this academic institution.

Variable	Contrast	Odds ratio	95% lower	95% upper	*p*-Value
Age	(Coefficient)	1.13	1.11	1.15	<0.001
Gender	Female–Male	1.14	0.99	1.32	0.073
Travel distance	(Coefficient)	0.99	0.99	1.00	0.221
SDI score	(Coefficient)	0.98	0.98	0.98	<0.001
Pandemic	Yes–No	3.15	2.47	4.02	<0.001
Payor	Self-pay public	0.92	0.80	1.06	0.260

## Data Availability

The data that support the findings of this study are available upon request from the corresponding author. The data are not publicly available due to privacy or ethical restrictions.

## References

[1] Dantas L. F., Fleck J. L., Cyrino Oliveira F. L., Hamacher S. (2018). No-Shows in Appointment Scheduling–A Systematic Literature Review. *Health Policy*.

[2] Discepolo K., Melvin P., Ghazarians M., Tennermann N., Ward V. L. (2023). Socioeconomic and Clinical Demography of Dental Missed Care Opportunities. *JDR Clinical & Translational Research*.

[3] McKernan S. C., Reynolds J. C., Ingleshwar A., Pooley M., Kuthy R. A., Damiano P. C. (2018). Transportation Barriers and Use of Dental Services Among Medicaid-Insured Adults. *JDR Clinical & Translational Research*.

[4] Flores G., Tomany-Korman S. C. (2008). The Language Spoken at Home and Disparities in Medical and Dental Health, Access to Care, and Use of Services in US Children. *Pediatrics*.

[5] Flores G., Lin H. (2013). Trends in Racial/Ethnic Disparities in Medical and Oral Health, Access to Care, and Use of Services in US Children: Has Anything Changed Over the Years?. *International Journal for Equity in Health*.

[6] Freeman R. (1999). Barriers to Accessing Dental Care: Patient Factors. *British Dental Journal*.

[7] Flores G., Abreu M., Brown V., Tomany-Korman S. C. (2005). How Medicaid and the State Children’s Health Insurance Program Can do a Better Job of Insuring Uninsured Children: The Perspectives of Parents of Uninsured Latino Children. *Ambulatory Pediatrics*.

[8] Mathu-Muju K. R., Li H. F., Hicks J., Nash D. A., Kaplan A., Bush H. M. (2014). Identifying Demographic Variables Related to Failed Dental Appointments in a University Hospital-Based Residency Program. *Pediatric Dentistry*.

[9] Wang N. J., Aspelund G. O. (2009). Children Who Break Dental Appointments. *European Archives of Paediatric Dentistry*.

[10] Social deprivation index (SDI) (2018). Robert Graham Center - Policy Studies in Family Medicine & Primary Care.

[11] GBD (2017). Global, Regional, and National Incidence, Prevalence, and Years Lived with Disability for 328 Diseases and Injuries for 195 Countries, 1990–2016: A Systematic Analysis for the Global Burden of Disease Study 2016. *The Lancet*.

[12] Peres M. A., Macpherson L. M. D., Weyant R. J. (2019). Oral Diseases: A Global Public Health Challenge. *The Lancet*.

[13] Office of Disease Prevention and Health Promotion (2030). Social Determinants of Health.

[14] Nelson T. M., Berg J. H., Bell J. F., Leggott P. J., Seminario A. L. (2011). Assessing the Effectiveness of Text Messages as Appointment Reminders in a Pediatric Dental Setting. *The Journal of the American Dental Association*.

[15] Awartani F. (2003). Broken Appointment Behavior in a Dental School Enviornment. *The Journal of Contemporary Dental Practice*.

[16] Iben P., Kanellis M. J., Warren J. (2000). Appointment-Keeping Behavior of Medicaid-Enrolled Pediatric Dental Patients in Eastern Iowa. *Pediatric Dentistry*.

[17] Almog D. M., Devries J. A., Borrelli J. A., Kopycka-Kedzierawski D. T. (2003). The Reduction of Broken Appointment Rates Through an Automated Appointment Confirmation System. *Journal of Dental Education*.

[18] Burger D. (2024). Buffalo Poised to Resume Fluoridation of City’s Water Supply. https://adanews.ada.org/ada-news/2024/january/buffalo-poised-to-resume-fluoridation-of-city-s-water-supply/.

[19] NIH (2024). Minority Health and Health Disparities: Definitions and Parameters. https://www.nimhd.nih.gov/about/strategic-plan/nih-strategic-plan-definitions-and-parameters.html.

[20] Lantos P. M., Hoffman K., Permar S. R. (2018). Neighborhood Disadvantage is Associated With High Cytomegalovirus Seroprevalence in Pregnancy. *Journal of Racial and Ethnic Health Disparities*.

[21] Kind A. J., Jencks S., Brock J. (2014). Neighborhood Socioeconomic Disadvantage and 30-Day Rehospitalization. *Annals of Internal Medicine*.

[22] Butler D. C., Petterson S., Phillips R. L., Bazemore A. W. (2013). Measures of Social Deprivation That Predict Health Care Access and Need Within a Rational Area of Primary Care Service Delivery. *Health Services Research*.

[23] Bevan G. H., Nasir K., Rajagopalan S., Al-Kindi S. (2022). Socioeconomic Deprivation and Premature Cardiovascular Mortality in the United States. *Mayo Clinic Proceedings*.

[24] Hijmans R. (2019). *Geosphere: Spherical Trigonometry*.

[25] Vincenty T. (1975). Direct and Inverse Solutions of Geodesics on the Ellipsoid With Application of Nested Equations. *Survey Review*.

[26] Burd C., Burrows M., McKenzie B. (2021). Travel Time to Work in the United States: 2019.

[27] Gelman A., Jakulin A., Pittau M. G., Su Y.-S. (2008). A Weakly Informative Default Prior Distribution for Logistic and Other Regression Models. *The Annals of Applied Statistics*.

[28] Gelman A., Carlin J. B., Stern H. S., Dunson D. B., Vehtari A., Rubin D. B. (2013). *Bayesian Data Analysis*.

[29] Huang Y., Hanauer D. A. (2014). Patient No-Show Predictive Model Development Using Multiple Data Sources for an Effective Overbooking Approach. *Applied Clinical Informatics*.

[30] Huang Y.-L., Hanauer D. A. (2016). Time Dependent Patient No-Show Predictive Modelling Development. *International Journal of Health Care Quality Assurance*.

[31] McLeod H., Heath G., Cameron E., Debelle G., Cummins C. (2015). Introducing Consultant Outpatient Clinics to Community Settings to Improve Access to Paediatrics: An Observational Impact Study. *BMJ Quality & Safety*.

[32] Torres O., Rothberg M. B., Garb J., Ogunneye O., Onyema J., Higgins T. (2015). Risk Factor Model to Predict a Missed Clinic Appointment in an Urban, Academic, and Underserved Setting. *Population Health Management*.

[33] Machado A. T., Werneck M. A., Lucas S. D., Abreu M. H. (2015). Who Did Not Appear? First Dental Visit Absences in Secondary Care in a Major Brazilian City: A Cross-Sectional Study. *Cien Saude Colet*.

[34] Storrs M. J., Ramov H. M., Lalloo R. (2016). An Investigation Into Patient Non-Attendance and Use of a Short-Message Reminder System at a University Dental Clinic. *Journal of Dental Education*.

[35] Ahluwalia K. P., Bessel D. R. (2010). Addressing Children’s Oral Health in Buffalo, New York.

[36] Wehby G. L., Shane D. M., Joshi A. (2017). The Effects of Distance to Dentists and Dentist Supply on Children’s Use of Dental Care. *Health Services Research*.

[37] Fisher-Owens S. A., Soobader M. J., Gansky S. A. (2016). Geography Matters: State-Level Variation in Children’s Oral Health Care Access and Oral Health Status. *Public Health*.

[38] Shariff J. A., Edelstein B. L. (2016). Medicaid Meets its Equal Access Requirement For Dental Care, But Oral Health Disparities Remain. *Health Affairs*.

[39] Lin M., Sappenfield W., Hernandez L. (2012). Child- and State-Level Characteristics Associated With Preventive Dental Care Access among U.S. Children 5–17 Years of Age. *Maternal and Child Health Journal*.

[40] Borrell L. N., Reynolds J. C., Fleming E., Shah P. D. (2023). Access to Dental Insurance and Oral Health Inequities in the United States. *Community Dentistry and Oral Epidemiology*.

[41] Flores G., Lin H., Walker C. (2017). The Health and Healthcare Impact of Providing Insurance Coverage to Uninsured Children: A Prospective Observational Study. *BMC Public Health*.

[42] Keisler-Starkey K., Bunch L. (2021). *Health Insurance Coverage in the United States: 2020*.

[43] McKernan S., Reynolds J., McQuistan M., Mascarenhas A. K., Okunseri C., Dye B. A. (2021). 3 - Access to Dental Care. *Burt and Eklund’s Dentistry, Dental Practice, and the Community*.

[44] Andersen R., Newman J. F. (1973). Societal and Individual Determinants of Medical Care Utilization in the United States. *The Milbank Memorial Fund Quarterly. Health and Society*.

